# No difference in mean middle cerebral artery blood velocity responses between lower‐ and upper‐body unilateral resistance exercise in untrained individuals

**DOI:** 10.1113/EP092859

**Published:** 2025-10-06

**Authors:** Stephanie Korad, Toby Mündel, Blake G. Perry

**Affiliations:** ^1^ School of Health Sciences Massey University Wellington New Zealand; ^2^ School of Sport, Exercise and Nutrition Massey University Palmerston North New Zealand; ^3^ Department of Kinesiology Brock University St Catharines Ontario Canada

**Keywords:** blood pressure, middle cerebral artery blood velocity, resistance exercise

## Abstract

Dynamic resistance exercise (RE) produces sinusoidal fluctuations in blood pressure that are mirrored by middle cerebral artery blood velocity (MCAv). However, whether lower‐ or upper‐body RE elicits a differential cerebrovascular response has not yet been examined. We investigated the cerebrovascular response to lower‐body RE versus upper‐body RE in 15 healthy untrained individuals (12 females and 3 males; mean ± SD; age 25 ± 6 years, height 179 ± 10 cm, weight 71 ± 15 kg and body mass index 24 ± 6 kg/m^2^). Participants completed four sets of 10 paced repetitions (15 repetitions/min) of unilateral leg‐extension exercise and unilateral bicep‐curl exercise at 60% of predicted one‐repetition maximum (leg extension 30 ± 9 kg and bicep curl 7 ± 3 kg). Beat‐to‐beat blood pressure, bilateral MCAv and partial pressure of end‐tidal carbon dioxide were measured throughout. Within‐exercise mean arterial blood pressure (MAP) and mean MCAv were averaged across the set. Additionally, zenith, nadir and zenith‐to‐nadir difference in MAP and mean MCAv for each repetition were averaged across each set. Baseline measures preceding each set were not different for all dependent variables, with no significant interaction differences observed (all *p >* 0.161). The mean MCAv within exercise decreased across sets (set effect *p <* 0.001), but MAP did not (*p = *0.071). No interaction effects were observed for any dependent variables (all *p *> 0.06), However, there was a zenith‐to‐nadir difference in mean MCAv (*p = *0.008), although *post hoc* tests revealed no significant difference between exercises (all *p >* 0.078). There were no differences in the cerebrovascular and cardiovascular responses to lower‐ and upper‐body RE, with similar sinusoidal fluctuations in MAP and MCAv_mean_ present during both exercises.

## Abstract (highlights)

- **What is the central question of this study?**
  Resistance exercise (RE) produces sinusoidal blood pressure fluctuations reflected in middle cerebral artery blood velocity. Comparing the blood pressure and cerebrovascular responses to lower‐ and upper‐body RE might reveal the haemodynamic impact of muscle mass recruitment to inform exercise selection for clinical populations in which transient elevation in blood pressure is contraindicated.
- **What is the main finding and its importance?**
  There was no difference in cerebrovascular responses to lower‐ and upper‐body RE in healthy untrained individuals, suggesting that during moderate‐intensity unilateral RE, exercise type and muscle mass might not determine the magnitude of fluctuations. Such RE might offer strength and vascular benefits without significant blood pressure elevations.

## 1 INTRODUCTION

Regular exercise is now viewed as an integral component of general health and well‐being. However, there are significant differences in the acute physiological responses and chronic adaptations to different exercise types. Although aerobic‐type exercise (e.g., running and cycling) produces modest changes in blood pressure, habitual exercise of this nature is associated with an increase in cerebral (Furby et al., 2019) and central arterial compliance (Tomoto et al., 2015), whereby those with high cardiorespiratory fitness demonstrate greater cerebrovascular reactivity to carbon dioxide (Smith et al., 2021). Conversely, several studies indicate that resistance exercise (RE), particularly at high intensity, might reduce central arterial compliance, both acutely after a single bout (DeVan et al., 2005) and chronically in RE‐trained individuals at rest (Miyachi, 2013; Miyachi et al., 2004). Resting cerebrovascular resistance is also increased following 12 weeks of RE but not following aerobic exercise (combination of running and cycling) interventions (Thomas et al., 2021). However, we have recently reported that RE‐trained individuals and untrained individuals have similar mean middle cerebral artery blood velocity (MCAv_mean_) responses to lower‐body RE, despite RE‐trained subjects having greater blood pressure perturbations during exercise (Korad et al., 2024a). Furthermore, RE‐trained individuals elicited pronounced decreases in blood pressure during standing post‐RE than their untrained counterparts but exhibited the same MCAv_mean_ and had similar recovery times (Korad et al., 2024b). When RE is combined with aerobic training it also generates a larger reduction in blood pressure variability in hypertensive patients (Caminiti et al., 2021). Thus, RE might yield potential benefits to the cerebral circulation by improving the buffering capacity to changes in perfusion pressure, although this is yet to be confirmed experimentally (Perry et al., 2019; Roy et al., 2022). Notwithstanding, RE yields many physiological benefits, including increases in muscular strength and increased muscle mass. However, the physiological adaptations to RE, particularly of the cardiovascular system, are not completely understood and must be considered for appropriate exercise prescription.

Evidence indicates that the within‐RE blood pressure response is determined by the muscle mass recruited and voluntary effort (Lewis et al., 1985; MacDougall et al., 1992, 1985); i.e., the larger the active muscle mass, the greater the pressor response. Moreover, during dynamic RE sinusoidal fluctuations in blood pressure are present, which reflect the phasic changes in muscle length (Perry & Lucas, 2021). Indeed, extremes in blood pressure have been observed during high‐intensity leg‐press exercise (i.e., 480 mmHg systolic and 350 mmHg diastolic pressures) (MacDougall et al., 1985). These perturbations in blood pressure are mirrored by concurrent changes in MCAv_mean_ (Edwards et al., 2002; Perry et al., 2014; Romero & Cooke, 2007), because the inherent lag in cerebral autoregulation generates a high‐pass filter system (Zhang et al., 1998). Modest RE‐induced perturbations in mean arterial blood pressure (MAP) and MCAv_mean_ can be produced during moderate‐intensity RE [60% of one‐repetition maximum (1RM)] when only a small muscle group/mass is recruited, during unilateral bicep curl (Korad et al., 2024c). It is worth noting that the magnitude of the rise in the rate pressure product is significantly reduced when bicep curls are performed unilaterally rather than bilaterally (Moreira et al., 2017). It is therefore plausible that moderate‐intensity, small‐muscle‐mass RE might still benefit the vasculature of the brain by provoking transient increases in perfusion and shear stress whilst avoiding the extreme increases in blood pressure.

Given the observed sinusoidal fluctuations in blood pressure and MCAv, it is possible that RE involving even a small muscle mass can generate a significant increase in shear stress. Previous studies examining the differences between lower‐ and upper‐body dynamic exercise used a cycle or arm ergometer and reported that upper‐body dynamic exercise led to higher blood pressure and heart rate than lower‐body dynamic exercise when performed at the same working intensities (Bobbert, 1960; Kang et al., 1999; Stenberg et al., 1967). However, these studies did not examine the cerebrovascular response to exercise, and to our knowledge, no data exist for comparing upper‐ and lower‐body RE exercise.Basic, moderate‐intensity, single joint RE could then be used to benefit clinical populations where exercise that is of high intensity, requires balance or stability, or requires a large muscle mass might be contraindicated or not possible (e.g., stroke or spinal cord injury).

To reduce variability related to training adaptations and better isolate acute cerebrovascular responses, studies need to focus exclusively on untrained individuals. Focusing on untrained individuals allows for controlling for potential cerebrovascular and autonomic adaptations associated with habitual RE training, which could confound acute response interpretation.

The aim of this study was to assess the haemodynamic response to upper‐ and lower‐body dynamic RE in healthy untrained individuals. The comparison between lower‐ and upper‐body RE is important owing to the differing cardiovascular and neuromuscular demands imposed by exercises involving distinct muscle groups and body regions. Lower‐body RE typically recruits a larger muscle mass and elicits greater systemic cardiovascular responses, including higher heart rate and blood pressure, in comparison to upper‐body RE. These physiological differences can distinctly influence cerebral perfusion and the regulation of MCAv. Perry et al. (2014) demonstrated an intensity‐dependent increase in MAP during lower‐body resistance exercise at 30%, 60% and 90% of 1RM. Notably, higher intensities elicited a more pronounced postexercise hypotensive response, which was accompanied by a proportional decrease in MCAv_mean_. In contrast, during upper‐body resistance exercise, Braz et al. (2014) reported a more modest MAP increase of ∼20 mmHg, alongside a 20%–24% rise in MCAv. Very few studies have directly compared upper‐ and lower‐body RE in terms of their cerebrovascular impact. This distinction is clinically and physiologically relevant, especially in populations where certain types of exercise might be prescribed preferentially (e.g., individuals with upper‐body function but lower‐limb impairment). Understanding whether upper‐ and lower‐body RE produce distinct cerebrovascular responses is crucial for informing safe and effective exercise prescription in both healthy individuals and those with compromised cerebrovascular function (e.g., post‐stroke or hypertensive populations). It might also help to identify whether one form of RE poses greater risk or benefit in terms of cerebral haemodynamic stability. We hypothesized that both lower‐ and upper‐body dynamic RE will produce sinusoidal fluctuations in blood pressure that will elicit similar cerebrovascular responses.

## 2 MATERIALS AND METHODS

### 2.1 Ethics and informed consent

All participants were fully informed of the experimental procedures, the purpose of the study and any potential risks associated with participation. Written informed consent was obtained from all participants before they took part in the research. The study was approved by the Massey University Human Ethics Committee (SOA 21/22) and adhered to the latest version of the *Declaration of Helsinki*, apart from registration in a database. This research was part of a broader study (Korad et al., 2024a, 2024b, 2024c) examining cerebrovascular responses to RE; however, all data presented here were collected independently and were not influenced by the additional aims or outcomes.

### 2.2 Participants

An a priori power analysis (G*Power v.3.1.9.4; Heinrich Heine University Düsseldorf, Düsseldorf, Germany) was conducted using data from the studies by Edwards et al. (2002) and Moralez et al. (2012) with similar interventions (dynamic resistance exercise), design and outcome measures (i.e., MCAv and MAP). The mean ± SD values for MCAv changes reported by Edwards et al. (2002) (mean increase of ∼10.5 ± 5.4 cm/s) and Moralez et al. (2012) (mean increase of ∼11.2 ± 4.8 cm/s) were used to estimate an expected effect size (Cohen's *d* ≈ 1.0). With an α‐level of 0.05 and a power (1 − *β*) of 0.80, the power analysis indicated that a minimum of 12 participants would be required to detect statistically significant within‐subject differences. A total of 15 participants (12 female) were recruited for this study (mean ± SD: age 25 ± 6 years, height 179 ± 10 cm, weight 71 ± 15 kg and body mass index 24 ± 6 kg/m^2^). All participants were healthy and free of any medical conditions, were not taking any form of medication other than oral contraception (*n* = 3) or an intrauterine device (*n* = 1), were non‐smokers and had no history or symptoms of cardiovascular, pulmonary, metabolic or neurological disease. Menstrual cycle phase was self‐reported by female participants, with all visits occurring during the early follicular phase (low oestrogen and progesterone) and during the placebo phase for those using oral contraceptives. Korad et al. (2022) and Favre and Serrador (2019) have previously reported no differences in functional cerebrovascular responses to acute changes in MAP and cerebral autoregulation between menstrual cycle phases. The participants were classified as healthy sedentary, which was determined by the following criteria: completing at most one dedicated exercise session per week for ≥6 months prior to the experiment. This does not include regular physical activity, e.g., activities that would be classified as low intensity, such as walking, gardening, low‐intensity cycling (commuting) and general household chores. If the participant engaged in rowing, they were excluded from the study, because rowing produces similar blood pressure fluctuations to RE (Pott et al., 1997). Untrained participants were selected because: (1) as discussed in the Introduction, although limited, there is evidence to indicate the habitual RE might subtly modify cerebrovascular function (Perry et al., 2019; Roy et al., 2022; Thomas et al., 2021); and (2) the results of the study could then inform prescription of RE for untrained populations.

### 2.3 Study design

All participants visited the temperature‐controlled laboratory three times, once for familiarization, then twice more for the experimental session for each exercise. A full explanation and demonstration of the risks of participation and the equipment and procedures used in the experiment were given during the familiarization session. Upon providing consent, the middle cerebral artery (MCA) contralateral to the exercising limb was insonated for the measurement of MCAv as described below. In addition, the participant's unilateral leg‐extension 1RM (dominant leg) and unilateral bicep‐curl 1RM (dominant arm) were estimated using the Brzycki (1993) expression: weight/[1.0278 − (0.0278 × number of repetitions)], and the working intensity for the trial, 60% of 1RM, was calculated (mean ± SD: leg extension predicted 1RM 52 ± 16 kg, 60% of 1RM 30 ± 9 kg; bicep curl predicted 1RM 9 ± 2 kg, 60% of 1RM 5 ± 1 kg). The participant also practised executing the leg extension and bicep curl at 60% of 1RM while maintaining the requested pacing and breathing pattern outlined below.

### 2.4 Experimental protocol

The experimental overview is shown in Figure 1. Briefly, the familiarization occurred >1 week before the trial, with subsequent experimental visits separated by >72 h. Participants arrived at the laboratory having refrained from consuming caffeine for 12 h and from vigorous exercise and alcohol consumption for ≥24 h prior. The participant was also instructed to consume 500 mL of water the night before and 500 mL ∼4 h before the experiment to ensure euhydration [urine specific gravity (USG) < 1.020].

**FIGURE 1:**
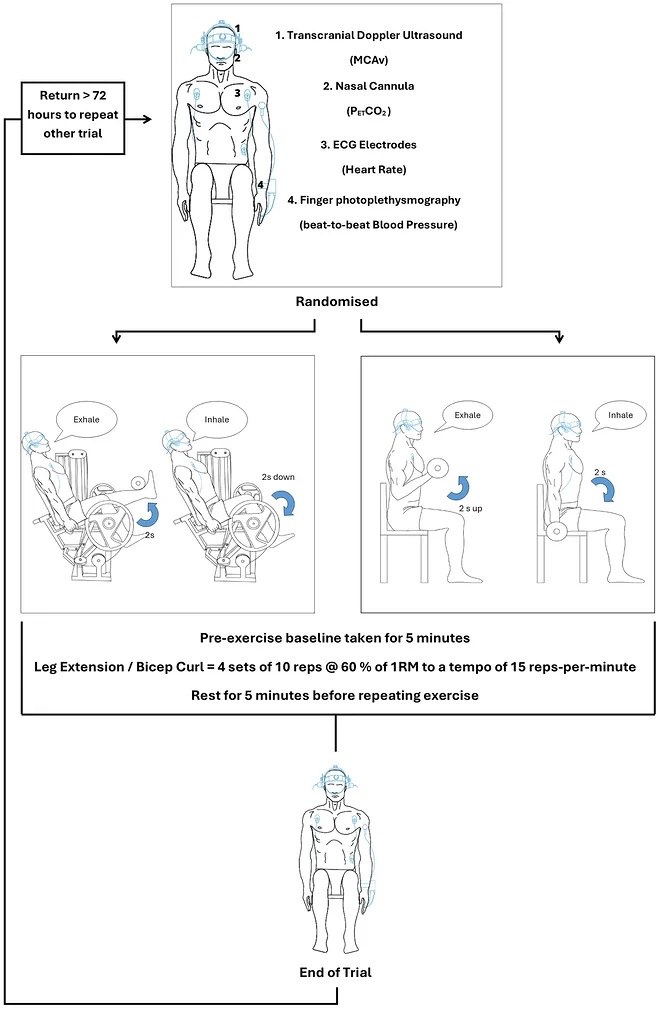
Experimental protocol. The exercise sets consisted of 10 repetitions of unilateral leg extensions or unilateral bicep curl at 60% of 1RM). Haemodynamic variables (MCAv, blood pressure and heart rate) and $P_{\mathrm{ETC}O_{2}}$ were measured throughout. Abbreviations: 1RM, one‐repetition maximum; MCAv, middle cerebral artery blood velocity; $P_{\mathrm{ETC}O_{2}}$, partial pressure of end‐tidal carbon dioxide.

On arrival, the participant was asked to provide a urine sample for USG analysis. The participant was then seated on a chair for instrumentation. Once instrumented, the participant rested quietly for 20 min for initial baseline recordings. For the leg‐extension trial, upon completion of baseline measures the participant was transferred to the leg‐extension machine, and baseline data were recorded for another 5 min. Data were recorded continuously throughout all trials, including between exercise bouts. During exercise, the participant performed 10 repetitions (reps) of unilateral leg extension or unilateral bicep curl at 60% of 1RM to a tempo of 15 reps‐per‐minute (timed by metronome), which equates to a repetition cycle length of 4 s (2 s per concentric and eccentric phase). An intensity of 60% of 1RM was chosen based on pilot testing to elicit characteristic sinusoidal fluctuations in blood pressure and MCAv while minimizing injury risk and avoiding the Valsalva manoeuvre. The Valsalva manoeuvre is typically used at intensities >80% of maximal voluntary contraction (MacDougall et al., 1992), and its recruitment would greatly confound the cerebrovascular response (Perry et al., 2014). Unilateral exercises targeting the dominant limb were selected to focus on contralateral middle cerebral artery responses owing to the decussation of motor tracts but also permitted the non‐invasive measurement of blood pressure in the inactive arm. Furthermore, using the dominant limb maximized participant comfort, coordination and movement consistency, reducing variability related to lack of familiarity in untrained individuals. The breathing sequence was matched to the tempo of the exercise, with exhalation during the concentric phase (2 s) and inhalation during the eccentric phase (2 s). This tempo was used to standardize the duration of muscular effort and increase time under tension. This deliberate pacing allowed for clearer observation of the expected sinusoidal fluctuations in both blood pressure and MCAv across the exercise cycle and allowed us to match ventilation with the tempo of the exercise. Participants were instructed to avoid the Valsalva manoeuvre during RE. Each exercise set was separated by 5 min of seated rest. A total of four sets of 10 reps were completed for each exercise. Four sets were used to replicate common resistance‐training practices, ensuring ecological validity and sufficient physiological stimulus to observe cerebrovascular changes across repeated bouts, with the latter confirmed during pilot testing. Each participant was reminded of the breathing technique prior to each set and reminded to avoid the Valsalva manoeuvre during exercise. The following criteria were used to ensure that the Valsalva manoeuvre was not performed.

1. No large and acute elevations in blood pressure were observed during exercise, beyond what would be expected for the prescribed intensity, as indicated by previous repetitions. Previous studies reported that when the Valsalva manoeuvre is recruited during resistance exercise the MAP (but not necessarily MCAv) is acutely elevated within the repetition (Perry et al., 2020, 2014).
2. Participants produced a capnograph that aligned with the paced breathing of the repetitions.
3. Participants were reminded of the exercise and breathing requirements for each set before and during each exercise bout.

### 2.5 Systemic haemodynamics

Heart rate (HR) was measured using a three‐lead ECG (ADInstruments, Australia). Non‐invasive beat‐to‐beat arterial blood pressure was measured by arterial volume clamp (Finapres Medical Systems, The Netherlands). The cuff was placed on the middle phalanx of either the middle finger or the index finger on the non‐dominant hand. The cuff was referenced to the level of the heart using the height correction unit. Blood pressure values were checked against an automated sphygmomanometer (Suresigns VM4, Philips Medical Systems, ‐ The Netherlands) during baseline and 2 min following each exercise bout and corrected when there was >5 mmHg difference in either systolic or diastolic blood pressures.

### 2.6 Middle cerebral artery blood velocity

Blood velocity in the MCA contralateral to the exercising limb was assessed using transcranial Doppler ultrasonography (Doppler‐Box X, DWL, Compumedics, Germany), which was sampled at a frequency of 1000 Hz. Transcranial Doppler data were visually inspected offline using DopplerX software. Segments with signal loss, motion artefacts or non‐physiological spikes were excluded. A minimum of three consecutive clean cardiac cycles were required for data inclusion, and trials with substantial data loss were excluded. To maintain signal stability, the probe was secured with an adjustable headband, and participants were instructed to minimize movement. The contralateral MCA was chosen owing to the decussation of motor tracts, because neurovascular coupling, the matching of cerebral perfusion to local neuronal activity, might lead to a greater increase in contralateral MCAv compared with the ipsilateral side, as seen in static hand‐grip exercise (Korad et al., 2024c). Blood velocity was measured in the M1 segment of the MCA using a 2 MHz probe, secured in place with an adjustable headband. The probe was positioned over the temporal window, above the zygomatic arch, following established search techniques (Aaslid et al., 1989; Willie et al., 2011). Ultrasound gel (Tensive, Parker Laboratory, Fairfield, NY, USA) was applied between the probe and the skin to ensure optimal signal quality. The average insonation depth of the MCA in this study was 54 ± 4 mm, consistent with previous findings by Bathala et al. (2013).

### 2.7 Partial pressure of end‐tidal carbon dioxide

The partial pressure of end‐tidal carbon dioxide ($P_{\mathrm{ETC}O_{2}}$) was measured using an online gas analyser (ML206 Gas Analyser, ADInstruments, Australia) and was collected throughout using a nasal cannula. The gas analyser was calibrated to a known gas concentration before each experiment.

### 2.8 Urine analysis

Hydration status has been reported to influence cerebrovascular regulation (Moralez et al., 2012; Perry et al., 2016); therefore, USG was used to confirm hydration status before each experiment using a hand‐held refractometer (Atago Co., Ltd, Tokyo, Japan). All participants were instructed to consume 500 mL of water the night before and 500 mL ∼4 h before the experiment. Approximately 30 min before the commencement of the experiment, USG was measured to confirm euhydration (mean ± SD 1.010 ± 0.007). If the participant did not meet the USG requirement, ∼500 mL of water was given to the participant and USG was remeasured 30 min after the consumption water, until a value of <1.020 was returned.

### 2.9 Data acquisition

All data were collected continuously using an analog‐to‐digital converter (PowerLab, ADInstruments, Australia) interfaced with a computer, then analysed using LabChart software (v.8.1.13 ADInstruments, Australia).

### 2.10 Data analysis

Mean MCAv (MCAv_mean_) was derived from the mean velocity of the raw MCAv waveform in LabChart. The MAP was calculated using the equation MAP = ⅓SBP + ⅔DBP, where SBP is the systolic blood pressure and DBP the diastolic blood pressure. The cerebrovascular conductance index (CVCi) was calculated using the equation CVCi = MCAv_mean_/MAP. The Gosling pulsatility index (PI) for the MCA was calculated as PI = SMCAv − DMCAv/MCAv_mean_ (Gosling & King, 1974), where SMCAv represents the maximum blood velocity in the MCA during systole, and DMCAv the minimum blood velocity in the MCA during diastole. Additionally, pulse pressure (PP) was calculated as SBP − DBP.

Given the sinusoidal haemodynamic profile during RE, the zenith and nadir MCAv_mean_ and MAP values were identified for each repetition, and the average values were calculated for each set. The zenith‐to‐nadir values for MCAv_mean_ and MAP were calculated by subtracting the nadir value from the zenith value of MCAv_mean_ and MAP, respectively, within each repetition. The average of these zenith‐to‐nadir values was then computed for each set, using methods described by Korad et al. (2024a).

Directional sensitivity of the cerebral pressure–flow relationship was quantified using time‐adjusted ratios of changes in MCAv_mean_ relative to MAP during transitions between concentric and eccentric muscular contractions (Labrecque et al., 2021) in the leg‐extension and bicep‐curl exercises. For each repetition, changes in MCAv_mean_ (ΔMCAv_T_) and MAP (ΔMAP_T_) were calculated separately for increases (concentric phase) and decreases (eccentric phase) in MCAv_mean_ and MAP, respectfully. The ratio was calculated as ΔMCAv_T_/ΔMAP_T_, as previously described by Labrecque et al. (2021). Additionally, the rates of change for MCAv_mean_ and MAP were determined by dividing the absolute change in each variable by the respective transition time during both MAP increases and decreases.

### 2.11 Statistical analysis

All data were analysed using SPSS statistical software v.28 (IBM Corp., Armonk, NY, USA). Statistical significance was set at *p ≤* 0.05. A two‐way ANOVA was performed to analyse baseline measures (exercise × baselines, 2 × 5) and dependent variables of interest during dynamic RE (exercise × sets, 2 × 4). *Post hoc* pairwise comparisons were used to isolate main effects in the data, and a Bonferroni correction factor was used when necessary. Partial η^2^ is reported for the interaction effect only, with large effect sizes identified as >0.1379, medium 0.0588–0.1379 and small <0.0588 (Cohen, 2013). A three‐way ANOVA was performed to examine directional sensitivity of the cerebral pressure–flow relationship. Phase (MAP increase vs. MAP decrease), exercise type (leg extension vs. bicep curl) and set (1–4) were the within‐subject factors. The dependent variables were the directional sensitivity ratio, rate of change in MAP and rate of change in MCAv. All data are displayed as the mean ± SD.

In addition to the ANOVA, Bayesian paired‐samples *t*‐tests were conducted to assess directly the strength of evidence for or against the null hypothesis of no difference between upper‐ and lower‐body RE across multiple physiological variables. Bayes factors (BF_10_) were calculated using JASP (v.0.19.3) with default priors (Cauchy prior width = 0.707). The BF_10_ values indicate the likelihood of the data under the alternative hypothesis relative to the null; values less than one support the null, and values greater than one support the alternative. BF_01_ (evidence for the null) was calculated as the inverse of BF_10_ (1/BF_10_). Interpretation thresholds followed Jeffreys (1961), with BF_01_ values between one and three considered anecdotal support for the null and three considered moderate support.

## 3 RESULTS

### 3.1 Baseline measurements

Baseline measures for the initial baseline immediately following instrumentation and the baseline prior to each set are shown in Table 1. There were no significant main effects of exercise or exercise × set interactions for any baseline values between exercises. Both exercise groups demonstrated equal ‘drift’ in the baseline variables.

**TABLE 1:** Lower‐ versus upper‐body baseline cerebrovascular and cardiovascular measures.

Variable	Exercise	Baseline period					*p*‐Value			Partial η^2^
		Initial	Prior to set 1	Prior to set 2	Prior to set 3	Prior to set 4	Exercise	Set	Interaction	
MCAv_mean_, cm/s	Lower body	69 ± 11	72 ± 11	71 ± 11	69 ± 11	70 ± 11^b^	0.141	<0.001	0.347	0.039
	Upper body	66 ± 11	66 ± 11	64 ± 11	63 ± 10^ab^	63 ± 10				
MAP, mmHg	Lower body	82 ± 10	86 ± 12	87 ± 13	87 ± 11	90 ± 12^a^, ^c^	0.445	<0.001	0.808	0.014
	Upper body	81 ± 6	84 ± 9^a^	86 ± 8^a^	85 ± 8^a^	88 ± 8^a^, ^b^, ^c^				
CVCi, cm/s/mmHg	Lower body	0.86 ± 0.14	0.86 ± 0.13	0.83 ± 0.14	0.81 ± 0.12	0.80 ± 0.14^b^, ^c^	0.055	<0.001	0.300	0.042
	Upper body	0.80 ± 0.14	0.76 ± 0.11^a^	0.73 ± 0.11^a^, ^b^	0.72 ± 0.12^a^, ^b^	0.70 ± 0.11^a^, ^b^				
PP, mmHg	Lower body	57 ± 13	57 ± 12	58 ± 14	57 ± 13	56 ± 14^c^	0.356	0.048	0.483	0.030
	Upper body	54 ± 12	54 ± 12	53 ± 12	52 ± 12	51 ± 13				
PI	Lower body	0.83 ± 0.18	0.79 ± 0.16	0.83 ± 0.16	0.84 ± 0.20	0.81 ± 0.16 ^d^	0.907	0.038	0.666	0.021
	Upper body	0.83 ± 0.14	0.82 ± 0.13	0.84 ± 0.15	0.83 ± 0.15	0.83 ± 0.16				
$P_{\mathrm{ETC}O_{2}}$, mmHg	Lower body	36 ± 4	37 ± 4	36 ± 4 ^b^	36 ± 4^b^	36 ± 4^b^	0.966	<0.001	0.364	0.038
	Upper body	37 ± 6	36 ± 5	36 ± 5	36 ± 5	36 ± 5				
HR, beats/min	Lower body	70 ± 16	70 ± 14	72 ± 15	70 ± 15	72 ± 15	0.841	0.544	0.161	0.056
	Upper body	71 ± 17	74 ± 15	71 ± 16	72 ± 15	72 ± 15				

*Note*: Data are presented the mean ± SD. Abbreviations: CVCi, cerebrovascular conductance index; HR, heart rate; MAP, mean arterial pressure; MCAv_mean_, middle cerebral artery blood velocity mean; $P_{\mathrm{ETC}O_{2}}$, end‐tidal partial pressure of carbon dioxide; PI; pulsatility index; PV, pulse velocity.

^a^ Different from initial.

^b^ Different from set 1.

^c^ Different from set 2.

^d^ Different from set 3.

### 3.2 Average response to dynamic resistance exercise

A typical response to RE is shown in Figure 2. The average cerebrovascular and cardiovascular responses within exercise are presented in Table 2. When examining the cerebrovascular response to the type of exercise, there were no significant exercise × set interaction differences between lower‐ and upper‐body SMCAv, DMCAv, MCAv_mean_, CVCi, or PI (*p*‐values all >0.081). Set differences were observed in the cerebrovascular variables SMCAv, DMCAv, MCAv_mean_ and CVCi and in the cardiovascular variables PP and $P_{\mathrm{ETC}O_{2}}$ (all *p* < 0.001), with decreases being seen across all variables.

**FIGURE 2:**
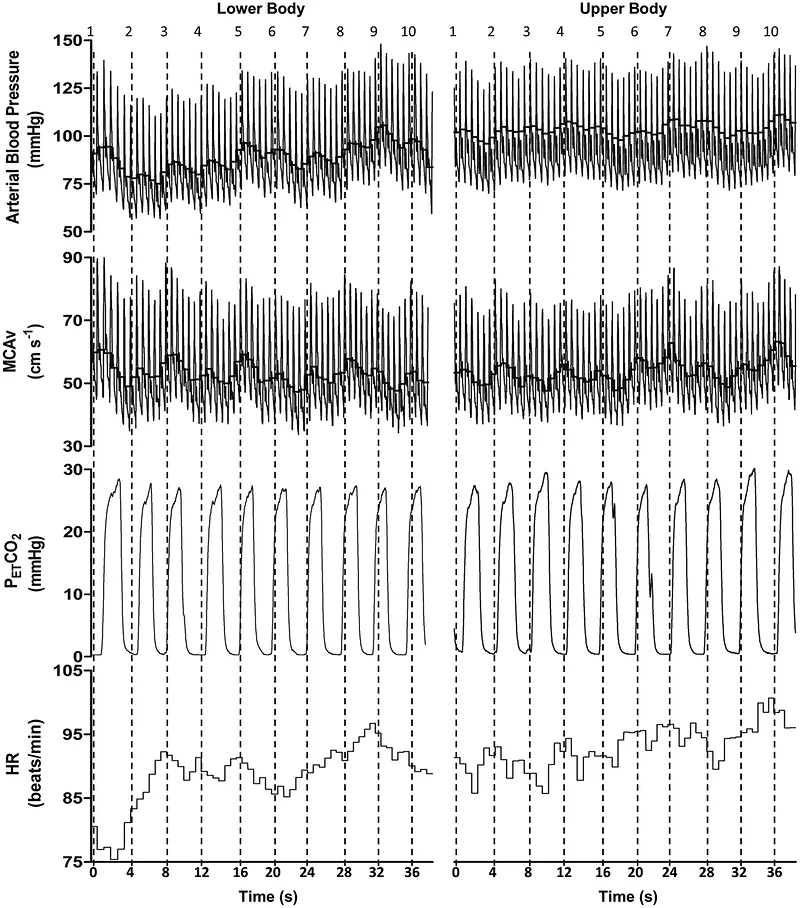
Typical trace of MCAv, arterial blood pressure, $P_{\mathrm{ETC}O_{2}}$ and HR during lower‐body (left) and upper‐body (right) resistance exercise for one participant. The thick black line in the MCAv and arterial blood pressure traces represent mean MCAv and mean arterial pressure, respectively. The numbers indicate the repetition count, with the dotted line denoting the start (concentric phase) of each repetition (*n* = 1). Abbreviations: HR, heart rate; MCAv, middle cerebral artery blood velocity;$P_{\mathrm{ETC}O_{2}}$, partial pressure of end‐tidal carbon dioxide.

**TABLE 2:** Averaged cerebrovascular and cardiovascular within exercise response during dynamic resistance exercise.

Variable	Exercise	Sets					*p*‐Value			Partial η^2^
		Initial	Set 1	Set 2	Set 3	Set 4	Exercise	Set	Interaction	
MCAv_mean_, cm/s	Lower body	69 ± 12	71 ± 11	67 ± 10^b^	65 ± 8^b^, ^c^	64 ± 8^b^, ^c^	0.328	<0.001	0.986	0.003
	Upper body	65 ± 12	66 ± 11	65 ± 10^b^	62 ± 10^b^	61 ± 10^b^				
MAP, mmHg	Lower body	81 ± 6	91 ± 12^a^	89 ± 9^a^	91 ± 9^a^	92 ± 9^a^	0.288	<0.001	0.124	0.062
	Upper body	82 ± 9	90 ± 9	90 ± 9	90 ± 9	94 ± 9^a^, ^c^				
CVCi, cm/s/mmHg	Lower body	0.85 ± 0.14	0.79 ± 0.13^a^	0.75 ± 0.11^a^	0.72 ± 0.10^a^, ^b^, ^c^	0.60 ± 0.11^a^, ^b^, ^c^	0.259	<0.001	0.958	0.006
	Upper body	0.80 ± 0.14	0.74 ± 0.13	0.71 ± 0.15	0.69 ± 0.12	0.66 ± 0.13^a^, ^b^, ^c^, ^d^				
PP, mmHg	Lower body	58 ± 13	56 ± 12	56 ± 13	53 ± 13^b^, ^c^	52 ± 12^b^, ^c^	0.299	<0.001	0.696	0.019
	Upper body	55 ± 12	51 ± 11	50 ± 11	49 ± 10^b^	48 ± 10^b^				
PI	Lower body	0.85 ± 0.18	0.80 ± 0.17	0.84 ± 0.16	0.82 ± 0.17	0.82 ± 0.15	0.734	0.291	0.839	0.013
	Upper body	0.84 ± 0.14	0.79 ± 0.14	0.80 ± 0.16	0.80 ± 0.14	0.80 ± 0.14				
$P_{\mathrm{ETC}O_{2}}$, mmHg	Lower body	36 ± 4	35 ± 4^a^	34 ± 4^a^	33 ± 4^a^	33 ± 4^a^	0.734	<0.001	0.930	0.008
	Upper body	37 ± 6	35 ± 5^a^	34 ± 5^a^	34 ± 5^a^	34 ± 4^a^				
HR, beats/min	Lower body	70 ± 16	91 ± 13^a^	88 ± 14^a^	88 ± 13^a^	89 ± 12^a^	0.701	<0.001	0.505	0.029
	Upper body	71 ± 17	87 ± 16^a^	92 ± 22^a^	92 ± 22^a^	92 ± 19^a^				

*Note*: Data are presented as the mean ± SD. Abbreviations: CVCi, cerebrovascular conductance index; HR, heart rate; MCAv_mean_, mean middle cerebral artery blood velocity; MAP, mean arterial blood pressure; $P_{\mathrm{ETC}O_{2}}$, end‐tidal partial pressure of carbon dioxide; PI, pulsatility index; PV, pulse velocity. *n* = 15.

^a^ Different from initial.

^b^ Different from set 1.

^c^ Different from set 2.

^d^ Different from set 3.

### 3.3 Bayesian paired‐sample *t*‐test for within‐exercise variables

To assess evidence for or against the null hypothesis (no difference between upper‐ and lower‐body RE) directly, Bayesian paired‐samples *t*‐tests were conducted. For MAP, Bayes factors (BF_10_) ranged from 0.268 to 0.313 across all sets, indicating moderate evidence for the null hypothesis (BF_01_ ≈ 3.2–3.7). Similar patterns were observed for HR (BF_10_ = 0.272–0.396; BF_01_ ≈ 2.5–3.7), $P_{\mathrm{ETC}O_{2}}$ (BF_10_ = 0.268–0.430; BF_01_ ≈ 2.3–3.7) and PI (BF_10_ = 0.262–0.661; BF_01_ ≈ 1.5–3.8), suggesting these variables were not meaningfully influenced by the exercising limb. In contrast, MCAv showed evidence for a difference during the early sets, with BF_10_ = 7.310 at Set 1 and BF_10_ = 3.024 at Set 2, indicating strong and moderate evidence, respectively, in favour of greater MCAv during upper‐body exercise. Likewise, PP showed moderate‐to‐strong evidence of a difference at Set 1 (BF_10_ = 4.719) and Set 2 (BF_10_ = 12.512). At later sets, BF_10_ values for MCAv and PP fell below two, suggesting a declining difference over time.

### 3.4 Zenith and nadir responses to dynamic resistance exercise

Similar to the average response to dynamic resistance exercise, there were no significant exercise × set interaction differences between the different modes of dynamic resistance exercise, as shown in Table 3 (all *p *> 0.136). However, set differences were observed for all cerebrovascular variables and cardiovascular variables during zenith and nadir except for SBP (all *p *> 0.084).

**TABLE 3:** Zenith and Nadir MCAv_mean_, MAP, CVCi, and zenith‐to‐nadir of MCAv_mean_ and MAP during dynamic resistance exercise.

Variable	Exercise	Sets				*p*‐Value			Partial η^2^
		Set 1	Set 2	Set 3	Set 4	Exercise	Set	Interaction	
Zenith									
SMCAv, cm/s	Lower body	107 ± 18	104 ± 17^b^	101 ± 15^b^, ^c^	99 ± 15^b^, ^c^	0.297	<0.001	0.375	0.036
	Upper body	101 ± 16	98 ± 16^b^	96 ± 15^b^	94 ± 15^b^, ^c^				
DMCAv, cm/s	Lower body	54 ± 8	50 ± 7^b^	50 ± 6^b^	49 ± 6^b^	0.363	<0.001	0.795	0.012
	Upper body	50 ± 9	48 ± 9	47 ± 9	47 ± 9				
MCAv, cm/s	Lower body	72 ± 10	68 ± 9^b^	67 ± 8^b^	66 ± 8^b^, ^c^	0.290	0.003	0.620	0.021
	Upper body	67 ± 11	64 ± 10^b^	63 ± 10^b^	62 ± 10^b^				
SBP, mmHg	Lower body	129 ± 22	124 ± 18	125 ± 18	126 ± 17	0.713	0.084	0.240	0.049
	Upper body	128 ± 17	126 ± 16	127 ± 17	132 ± 17				
DBP, mmHg	Lower body	75 ± 10	73 ± 9	76 ± 10	77 ± 9^c^	0.729	0.006	0.651	0.019
	Upper body	75 ± 9	75 ± 8	76 ± 9	79 ± 10				
MAP, mmHg	Lower body	93 ± 13	90 ± 11	92 ± 11	93 ± 11^c^	0.683	0.020	0.398	0.034
	Upper body	93 ± 10	92 ± 10	93 ± 9	99 ± 11				
CVCi, cm/s/mmHg	Lower body	0.78 ± 0.14	0.78 ± 0.14	0.74 ± 0.12	0.72 ± 0.13^b^, ^c^	0.202	<0.001	0.833	0.010
	Upper body	0.74 ± 0.12	0.71 ± 14	0.69 ± 0.12^c^	0.66 ± 0.13^b^, ^c^, ^d^				
Nadir									
SMCAv, cm/s	Lower body	103 ± 17	99 ± 18	96 ± 15^b^	94 ± 15^b^, ^c^	0.278	<0.001	0.189	0.055
	Upper body	95 ± 16	92 ± 16^b^	91 ± 15^b^	89 ± 15^b^				
DMCAv, cm/s	Lower body	48 ± 8	45 ± 6^b^	44 ± 6^b^	43 ± 6^b^	0.276	<0.001	0.143	0.062
	Upper body	43 ± 9	42 ± 8^b^	41 ± 8^b^	41 ± 8^b^				
MCAv, cm/s	Lower body	66 ± 10	63 ± 9^b^	61 ± 7^b^	60 ± 8^b^	0.236	<0.001	0.136	0.063
	Upper body	60 ± 11	59 ± 10^b^	58 ± 10^b^	57 ± 10^b^				
SBP, mmHg	Lower body	125 ± 22	119 ± 17	120 ± 17	120 ± 16	0.854	0.140	0.148	0.066
	Upper body	121 ± 16	120 ± 16	121 ± 16	126 ± 14				
DBP, mmHg	Lower body	71 ± 10	69 ± 9	71 ± 9	72 ± 8	0.773	0.027	0.479	0.029
	Upper body	70 ± 8	70 ± 7	72 ± 7	74 ± 9				
MAP, mmHg	Lower body	89 ± 13	86 ± 10	87 ± 10	88 ± 10	0.784	0.050	0.207	0.052
	Upper body	87 ± 9	87 ± 8	88 ± 8	92 ± 9^c^				
CVCi, cm/s/mmHg	Lower body	0.75 ± 0.1	0.74 ± 0.14	0.70 ± 0.12	0.69 ± 0.13^c^	0.199	<0.001	0.900	0.007
	Upper body	0.69 ± 0.1	0.68 ± 0.14	0.65 ± 0.12	0.62 ± 0.12^b^, ^c^, ^d^				
Zenith‐to‐nadir difference									
MCAv_mean_, cm/s	Lower body	5 ± 1	5 ± 2	5 ± 2	6 ± 2	0.613	0.069	0.008^*^	0.130
	Upper body	7 ± 2	6 ± 2	6 ± 2	5 ± 2				
MAP, mmHg	Lower body	4 ± 2	4 ± 2	5 ± 2	5 ± 2	0.362	0.301	0.098	0.072
	Upper body	6 ± 2	5 ± 2	5 ± 2	5 ± 2				

*Note*: Data are presented as the mean ± SD. Abbreviations: CVCi, cerebrovascular conductance index; DBP, diastolic blood pressure; DMCAv, diastolic middle cerebral artery blood velocity; MAP, mean arterial blood pressure; MCAv_mean_, mean middle cerebral artery blood velocity; SBP, systolic blood pressure; SMCAv, systolic middle cerebral artery blood velocity.

^b^ Different from set 1.

^c^ Different from set 2.

^d^ Different from set 3.

* Interaction effect, but *post hoc* test revealed no differences.

### 3.5 Zenith‐to‐nadir difference

No significant effect of exercise was observed in MCA_vmean_ (*p* = 0.613) or for MAP (*p* = 0.362). However, an exercise × set interaction effect was seen in MAP (*p* = 0.008); however, paired *t*‐test revealed no significant differences between exercise types.

### 3.6 Directional sensitivity ratio and rate of change

Table 4 presents the directional sensitivity ratios (ΔMCAv_T_/ΔMAP_T_) and rates of change for increases and decreases in MAP and MCAv_mean_ across four sets of both lower‐ and upper‐body RE. No significant differences were observed in directional sensitivity ratios between exercise types (lower vs. upper body) or across sets for either increases (*p* = 0.892 and *p* = 0.689 for exercise and set interaction effects, respectively) or decreases (*p* = 0.881 and *p* = 0.828). Likewise, the rates of change in MAP and MCAv_mean_ during both increases and decreases showed no significant different across exercise type or sets (see Table 4).

**TABLE 4:** Directional sensitivity ratios and rate of change for increases and decreases in MAP and MCAv_mean_.

Variable	Exercise	Sets				*p*‐Value			Partial η^2^
		Set 1	Set 2	Set 3	Set 4	Exercise	Set	Interaction	
ΔMCAv_T_/ΔMAP_Tincrease_	Lower body	2.1 ± 1.0	2.0 ± 0.9	1.8 ± 1.0	1.8 ± 1.0	0.892	0.689	0.279	0.044
	Upper body	1.8 ± 0.7	1.9 ± 0.8	1.9 ± 0.6	1.8 ± 0.7				
ΔMCAv_T_/ΔMAP_Tdecrease_	Lower body	2.2 ± 1.0	2.0 ± 1.0	2.1 ± 1.4	1.7 ± 0.8	0.881	0.828	0.236	0.049
	Upper body	1.9 ± 0.7	1.9 ± 0.9	1.9 ± 0.7	2.1 ± 1.1				
Rate of change MAP_Tincrease_, mmHg/s	Lower body	2.9 ± 1.2	2.8 ± 1.3	3.2 ± 1.1	3.0 ± 1.0	0.472	0.452	0.630	0.020
	Upper body	2.7 ± 0.8	2.7 ± 0.9	2.7 ± 1.0	2.8 ± 1.1				
Rate of change MAP_Tdecrease_, mmHg/s	Lower body	−2.4 ± 1.2	−2.4 ± 1.1	−2.8 ± 1.2	−2.8 ± 1.1	0.609	0.103	0.358	0.037
	Upper body	−2.5 ± 0.8	−2.2 ± 0.7	−2.3 ± 0.9	−2.7 ± 1.4				
Rate of change MCAv_Tincrease_, cm/s^2^	Lower body	4.1 ± 1.0	3.8 ± 1.4	4.1 ± 1.3	3.9 ± 1.5	0.458	0.108	0.889	0.007
	Upper body	3.9 ± 1.5	3.5 ± 1.3	3.8 ± 1.1	3.4 ± 1.1				
Rate of change MCAv_Tdecrease_, cm/s^2^	Lower body	−3.7 ± 2.0	−3.8 ± 2.4	−3.6 ± 3.0	−3.5 ± 1.9	0.909	0.444	0.796	0.012
	Upper body	−4.0 ± 1.5	−3.7 ± 1.4	−3.6 ± 1.3	−3.6 ± 1.7				

*Note*: Data are presented as the mean ± SD, Abbreviations: MAP, mean arterial blood pressure; MCAv, middle cerebral artery blood velocity. The directional sensitivity ratio is the proportion of ΔMCAv_T_/ΔMAP_T_ in each direction. The MCAv_T_ rate of change is the proportion of ΔMCAv/ΔTimeMCAv in each direction. The MAP_T_ rate of change is the proportion of ΔMAP/ΔTimeMAP in each direction.

The three‐way repeated‐measures ANOVA on directional sensitivity ratio, MAP_T_ rate of change and MCAv_T_ revealed that there was a main effect of set for all three outcomes (all *p < *0.001). There was no significant main effect of exercise type (*F* = 1.029, *p = *0.328) or phase (*F* = 1.822, *p = *0.199) and no significant two‐ or three‐way interactions (all *p > *0.299). Likewise, for MAP_T_ rate of change and MCAv_T_ rate of change, there was also no significant main effect of exercise (MAP_T_: *F* = 0.419, *p = *0.495; MCAv_T_: *F* = 0.001, *p = *0.982) or phase (*F* = 2.095, *p = *0.170 and *F* = 1.182, *p = *0.295, respectively). Furthermore, there were no significant two‐ or three‐way interactions for MAP_T_ rate of change and MCAv_T_ rate of change (all *p > *0.194).

## 4 DISCUSSION

The purpose of this study was to investigate the haemodynamic responses to lower‐ and upper‐body dynamic RE in healthy untrained individuals. In agreement with our hypothesis, the data revealed no significant differences between the cardiovascular or cerebrovascular responses to lower‐ and upper‐body dynamic RE. The findings of the study indicate that during moderate‐intensity unilateral dynamic RE, both upper‐ and lower‐body exercises produced similar modest perturbations in MAP and MCAv_mean_.

Both lower‐ and upper‐body RE elicited modest sinusoidal fluctuations in blood pressure, which were reflected in similar MCAv_mean_ responses, suggesting that in the present experiment the muscle mass used during dynamic RE did not determine the haemodynamic response. Other studies have demonstrated that RE with a larger muscle mass produces greater increases in blood pressure (Lewis et al., 1985; MacDougall et al., 1985). MacDougall et al. (1985) did report that at the same intensity (95% of 1RM to fatigue), the absolute blood pressures were greater in the single leg press than the single arm bicep curl. However, data were reported only for five well‐RE‐trained males. Others have also reported significantly larger perturbations in blood pressure at similar intensities to that of the present study during upright squatting (Perry et al., 2014). Moreover, body weight squats (i.e., with no additional load) produce pronounced fluctuations in blood pressure, and subsequently, MCAv_mean_ (Labrecque et al., 2021). These ‘forced’ oscillations in blood pressure are typically completed at frequencies of 0.05 and 0.10 Hz and used experimentally to assess dynamic cerebral autoregulation. However, given that the RE‐induced 0.25 Hz oscillations in the present study are faster than those typically used experimentally, it is likely that these perturbations occur at a frequency where dynamic cerebral autoregulation is less effective (e.g., >0.20 Hz) (Claassen et al., 2009, 2016; Panerai et al., 2023). Burma et al. (2024), demonstrated that directional sensitivity varies across phases of the cardiac cycle and is influenced by oscillation frequency, emphasizing why RE‐induced fluctuations might produce different responses from other methods of forcing blood‐pressure oscillations e.g., sit‐to‐stand manoeuvre. Other exercises (e.g., Olympic style lifting) that also possess an orthostatic component exacerbate the changes (both increases and decreases) in blood pressure and challenge cerebral perfusion regulatory mechanisms (i.e., cerebral autoregulation) during and after RE (Compton et al., 1973), whereas an exercise such as a leg press, where the feet are above heart level, does not provide this orthostatic stress (Edwards et al., 2002). Therefore, muscle mass might not be the only factor determining the blood pressure response to RE, but a combination of body position, type of exercise, recruited muscle mass and the Valsalva manoeuvre (Perry & Lucas, 2021). Further research involving the comparison of bilateral RE with larger muscle mass (e.g., bilateral bicep curl vs. bilateral leg press) across a variety of body positions is required to confirm the findings of the present study.

We have previously shown more effective MCAv regulation during RE in RE‐trained individuals (Korad et al., 2024a), whereas others have shown that RE‐trained individuals lack a hysteresis‐like pattern in cerebral autoregulation during forced oscillations in MAP generated by repeated squat–stand manoeuvres (Roy et al., 2022). Labrecque et al. (2026) also quantified directional sensitivity during repeated squat–stand protocols, finding substantial variability depending on oscillation duration and thereby strengthening the notion that RE adaptations to cerebrovascular function might be task or frequency specific. The present study revealed a main effect of set for directional sensitivity ratio and rates of change for MAP and MCAv, indicating that directional sensitivity ratio, MAP rate of change and MCAv rate of change changed significantly over successive sets. However, there was no main effect of exercise type or phase nor significant two‐ and three‐way interactions. These findings indicate that although there were changes across sets, cerebrovascular autoregulatory capacity was preserved and did not differ between upper‐ and lower‐body RE during moderate‐intensity RE. These findings align with recent work by Allison et al. (2025), who found that successive high‐intensity leg‐press exercise at 90% of 1RM did not affect the directional sensitivity of the cerebral pressure–flow relationship or its components. Furthermore, the authors reported that successive sets of high‐intensity leg‐press exercise at 90% of 1RM does not significantly alter MAP or MCAv responses during or immediately after exercise, indicating stable cerebral and systemic haemodynamics. A study investigating the effects of high‐intensity interval training to exhaustion on directional sensitivity reported that high‐intensity interval training potentially influences directional sensitivity (Abbariki et al., 2022). The authors found that directional sensitivity disappeared at 0.10 Hz MAP oscillations, and during 0.05 Hz MAP oscillations there was an inverted directionality pattern, highlighting that intensity might play an influential role on directional sensitivity.

It is important to note that high‐intensity RE, especially when the Valsalva manoeuvre is used, can induce maladaptation, such as increased central arterial compliance (Miyachi, 2013) and increased resting cerebrovascular resistance following 12 weeks of RE (Thomas et al., 2021). However, given only moderate‐intensity and small‐muscle‐mass exercises used herein, we report only modest perturbations in blood pressure and MCAv, which might be insufficient to generate such maladaptation if performed regularly. Indeed, the small unilateral muscle mass RE used in the present study could potentially benefit those with limited exercise capacity.

Shear stress is integral to endothelial health and vascular function, with transient increases in shear stress having anti‐atherogenic effects (Green et al., 2017). Aerobic exercise has been demonstrated to increase internal carotid artery and vertebral artery shear (Smith et al., 2019) and might improve cerebral endothelial function (Sakamoto et al., 2024). Although not as marked as during high‐intensity treadmill running, endothelial shear stress in the common carotid artery is increased during bicep curls (Montalvo et al., 2022). Given the pattern of sinusoidal fluctuations in blood pressure and MCAv observed (see Figure 2), it is possible that RE even with a small muscle mass might produce a significant increase in shear within the extracranial feed arteries and the cerebral circulation. Hypothetically, the subsequent transient increase in cerebrovascular shear, with only modest fluctuations in blood pressure, could be beneficial to clinical populations who are unable to perform aerobic exercise or bilateral RE (e.g., stroke patients with hemiplegia or hemiparesis).

Current evidence indicates that RE could be beneficial in the recovery from stroke (Billinger et al., 2014; Veldema & Jansen, 2020), and although the strength and hypertrophy improvements following RE training are the main physiological adaptations, it is plausible that vascular function could also be improved. However, further research is required to confirm this, because the MCAv response to recumbent stepping (e.g., aerobic exercise) at moderate (Kempf et al., 2019) and high (Whitaker et al., 2024) intensities is blunted in stroke patients.

### 4.1 Limitations

We have previously reported greater within‐RE blood pressures in RE‐trained individuals compared with RE untrained individuals (Korad et al., 2024a). Given that the present study participants did not habitually engage in RE, further research is required to investigate the haemodynamic responses to upper‐ and lower‐body RE in RE‐trained individuals, because the within‐exercise blood pressures might exceed those reported here. Moreover, the present study used basic single‐joint exercises that recruited small muscle groups unilaterally. These exercises were selected because: (1) they are easy to perform and do not require extensive RE training/experience and were therefore appropriate for the untrained cohort that participated in the present study; and (2) the unilateral nature permitted the measurement of continuous blood pressure via finger photoplethysmography. Therefore, the present findings are limited to the RE used herein, and further research is required to compare the effects of upper‐ and lower‐body RE that is bilateral in nature, recruits a larger muscle mass, adopts a different body position [semi‐recumbent (e.g., leg press) or supine (bench press)] and/or produces more pronounced perturbations in blood pressure.

Although not intentional, a greater proportion of female participants completed the present study. Males typically exhibit a more pronounced exercise pressor response, characterized by greater increases in arterial blood pressure during static hand‐grip exercise compared with females (Ettinger et al., 1996; Matthews & Stoney, 1988; Simoes et al., 2013). However, previous research exploring the effect of sex on the haemodynamic responses to dynamic exercise has shown that when factors such as body surface area, body composition (Bassareo & Crisafulli, 2020), body size and strength are statistically comparable between sexes, differences in the exercise pressor reflex are minimized or absent (Tharpe et al., 2023). Regarding the regulation of cerebral blood flow, there is some evidence that cerebral autoregulation is improved in females during forced oscillations in blood pressure produced by repeated squat–stand and sit‐to‐stand manoeuvres (Favre & Serrador, 2019). However, Labrecque, Rahimaly et al. (2019) challenge that notion and reported that dynamic cerebral autoregulation is attenuated in aerobically fit women. The discrepancies in results might be accredited to the training status of the participants included in the studies (Labrecque, Smirl et al., 2019). A meta‐analysis by Skinner et al. (2021), and subsequently, a more recent study by Korad et al. (2022), found no significant differences in cerebral autoregulation across the phases of the menstrual cycle. Furthermore, Labrecque et al. (2022) assessed the reproducibility and diurnal variation of directional sensitivity and reported that these metrics are both stable throughout the day and unaffected by biological sex, reinforcing that directional sensitivity is robust under repeated MAP oscillations. This conclusion was challenged by Allison et al. (2025), who found that during the oscillations in MAP during the leg‐press exercise, there were significantly greater changes in MCAv during the midluteal phase of the menstrual cycle versus the early follicular phase. Furthermore, MCAv_mean_, maximum MCAv, minimum MCAv and MAP minimum and maximum were higher in the midluteal phase compared with the early follicular phase. Further studies are needed to examine the difference in menstrual cycle phase and cerebrovascular response to resistance exercise. Although there is some evidence to indicate subtle differences in cardio‐ and cerebrovascular regulation between sexes, given that the present experiment is a within‐subject design, the effects of sex are largely mitigated. However, further research is warranted to investigate the effect of sex on the cerebrovascular responses to RE.

When interpreting the results of this study, it is important to acknowledge certain methodological limitations. In this study, MCAv was measured via transcranial Doppler as a proxy for cerebral blood flow. However, this proxy operates on the assumption that the diameter of the MCA is unchanged (Ainslie & Hoiland, 2014). The observed decrease in MCAv_mean_ might indeed be influenced by a reduction in $P_{\mathrm{ETC}O_{2}}$, which could be affected by the paced breathing pattern adopted during RE. Indeed, pre‐exercise hyperventilation reduces within‐RE average MCAv across the set (Romero & Cooke, 2007). Although a causative relationship cannot be established definitively within the present study design, it is reasonable to speculate, given the well‐documented sensitivity of cerebral blood flow to changes in arterial CO_2_, that fluctuations in $P_{\mathrm{ETC}O_{2}}$ are likely to have contributed to the observed changes in MCAv_mean_. Previous research suggests that MCA diameter can change in certain physiological conditions. Verbree et al. (2014) reported no change in MCA diameter during mild hypocapnia (a 7.5 mmHg reduction in $P_{\mathrm{ETC}O_{2}}$), whereas Coverdale et al. (2014) reported that the real decrease in cerebral blood flow during more severe hypocapnia ($P_{\mathrm{ETC}O_{2}}$ of ∼23 mmHg) was 7% ± 4% greater than the transcranial Doppler‐reported MCAv. Only small decreases in $P_{\mathrm{ETC}O_{2}}$ (∼1–2 mmHg) were observed during RE in the present study, owing to the implementation of paced breathing, and this was unlikely to have generated a significant change in MCA diameter. However, Verbree et al. (2017), using high‐resolution MRI, reported a 2% reduction in MCA cross‐sectional area during simple hand‐grip exercise, potentially attributable to sympathetic vasoconstriction. Therefore, the findings of the present study should be interpreted with caution.

## 5 CONCLUSION

The findings of the present study indicate that there were no significant differences in MCAv_mean_ between lower‐ and upper‐body unilateral dynamic RE. The absence of a difference in MCAv_mean_ between upper‐ and lower‐body RE might be attributed to the lack of differences observed in MAP between conditions. Further research is needed to determine whether increasing exercise intensity, shifting from unilateral to bilateral movements or altering body position would elicit measurable changes in cerebrovascular responses (i.e., shear) and be beneficial for clinical populations.

## AUTHOR CONTRIBUTIONS

Stephanie Korad, Toby Mündel and Blake Perry conceptualized and designed the research. Stephanie Korad and Blake Perry were responsible for data collection. Stephanie Korad, Toby Mündel and Blake Perry were responsible for data analysis, interpretation and drafting of the article. All authors have read and reviewed the article and provided critical feedback. All authors have approved the final version of this manuscript and agree to be accountable for all aspects of the work in ensuring that questions related to the accuracy or integrity of any part of the work are appropriately investigated and resolved. All persons designated as authors qualify for authorship, and all those who qualify for authorship are listed.

## CONFLICT OF INTEREST

The authors have nothing to report.

## Data Availability

The data that support the findings of this study are available from the corresponding author upon reasonable request.
